# Reciprocal Regulation of Development of Neutrophil-Dendritic Cell Hybrids in Mice by IL-4 and Interferon-Gamma

**DOI:** 10.1371/journal.pone.0082929

**Published:** 2013-11-21

**Authors:** Shuo Geng, Hironori Matsushima, Takashi Okamoto, Yi Yao, Ran Lu, Akira Takashima

**Affiliations:** Department of Medical Microbiology and Immunology, University of Toledo College of Medicine, Toledo, Ohio, United States of America; Istituto Superiore di Sanità, Italy

## Abstract

Neutrophils contribute to innate host immunity by functioning as professional phagocytes, whereas dendritic cells (DCs) are prototypic antigen presenting cells (APCs) responsible for the induction of adaptive immune responses. We have demonstrated recently that neutrophils trans-differentiate into a unique population, termed “neutrophil-DC hybrids,” expressing surface markers of both neutrophils and DCs and exhibiting dual functionality of both phagocytes and APCs. Although the hybrid cells emerged in significant numbers in murine bone marrow (BM) culture in the presence of GM-CSF, mechanisms regulating their development remained mostly unknown. In this study, we tested a total of 61 cytokines for their potentials to regulate neutrophil-DC hybrid formation using a newly developed BM micro-culture system combined with semi-automated FACS analysis. Several cytokines including GM-CSF were found to promote the generation of neutrophil-DC hybrids defined by the phenotype of CD11c^+^/MHC II^+^/Ly6G^+^. When tested in the presence of GM-CSF, hybrid cell development was enhanced by IL-4 and suppressed by interferon-γ (IFNγ) in dose-dependent fashions. We next determined in vivo impacts of IL-4 and IFNγ on the development of neutrophil-DC hybrids in thioglycollate-induced peritonitis lesions. Intraperitoneal administrations of IL-4/anti-IL-4 antibody complex (IL-4C) significantly increased the number of hybrids recovered from the lesions. By contract, recovery of hybrids was reduced by recombinant IFNγ. With regard to function, those hybrid cells recovered from IL-4C-treated mice and IFNγ-treated mice showed potent abilities to capture *E.coli*. These observations imply that emergence of neutrophil-DC hybrids in inflammatory sites is tightly regulated by local cytokine milieus.

## Introduction

Large numbers of neutrophils are constantly released from bone marrow (BM) into blood circulation. Neutrophils are generally regarded as terminally differentiated leukocytes destined to undergo apoptosis in the steady state or to function as professional phagocytes under pathological conditions. Once recruited to the sites of injury or infection, neutrophils amplify inflammatory responses by releasing cytokines, chemokines, and chemical mediators, clear invading microorganisms by releasing anti-microbial agents and by extruding neutrophil extracellular traps (NETs), and remodel damaged tissues by digesting tissue debris [1]. Recent studies have suggested that neutrophils may also participate in adaptive immunity [2]. For example, neutrophils can induce the maturation of dendritic cells (DCs) [3], recruit T cells to inflamed tissues [4], and promote immunoglobulin class-switching and antibody production by B cells [5]. When cultured in the presence of selected cytokines, neutrophils can even acquire surface expression of MHC class II molecules and other markers of professional antigen presenting cells (APCs) [6–10]. Furthermore, unusual neutrophils expressing APC markers have been detected in experimentally induced colitis lesions in mice [11], synovial fluid samples from rheumatoid arthritis patients [12,13], and peripheral blood samples from autoimmune vasculitis patients [14,15]. 

 We have recently reported that when cultured in the presence of GM-CSF, Gr-1^high^/CD48^−^ immature neutrophils purified from mouse BM differentiate into a unique leukocyte population, termed “neutrophil-DC hybrid,” expressing surface markers of both neutrophils (Ly6G, 7/4, CD62L, and CXCR2) and DCs (CD11c, MHC II, CD80, and CD86). Neutrophil-DC hybrids retain the functionality of professional phagocytes to incorporate soluble and particulate materials efficiently, to extrude NETs upon stimulation, and to kill bacteria rapidly in a cathelicidin-dependent fashion. At the same time, they exhibit key functionality of APCs to present various forms of antigen to immunologically naïve CD4 T cells [16]. With regard to in vivo relevance, only few neutrophil-DC hybrids were found in any of the tested organs. However, they became readily detectable in the skin, lung, lymph nodes, and peritoneal cavity under inflammatory conditions. For example, CD11c^+^/MHC II^+^/Ly6G^+^ hybrids emerged in thioglycollate-induced peritonitis lesions in a time-dependent fashion with a peak observed on Day 2. Those hybrids emerging at inflammatory sites also exhibited dual functionality to serve as both professional phagocytes and APCs –they cleared bacteria rapidly and presented bacterial antigen to CD4 T cells efficiently [17]. Not only do these findings demonstrate striking plasticity of neutrophils, they also suggest that neutrophil-DC hybrids may play protective roles against bacterial infection and perhaps pathogenic roles in autoimmune inflammatory diseases. One of the key questions concerns mechanisms regulating neutrophil transdifferentiation into hybrids. Although we have found that GM-CSF can promote neutrophil transdifferentiation into hybrids [16], it remains unknown whether development of neutrophil-DC hybrids is regulated by other cytokines. The objective of the present study was, therefore, to screen a variety of cytokines for their potentials to promote or suppress hybrid cell development in an unbiased, systematic fashion. 

## Methods

### A cytokine library

We prepared a test library by purchasing the following murine recombinant cytokines and chemokines (61 in total) from PeproTech (Rocky Hill, NJ): interleukin (IL)-1α, IL-1β, IL-2, IL-3, IL-4, IL-5, IL-6, IL-7, IL-9, IL-10, IL-11, IL-12, IL-13, IL-15, IL-17A, IL-17F, IL-18, IL-21, IL-22, IL-31, IL-33, cardiotrophin-1 (CT-1), G-CSF, M-CSF, GM-CSF, stem cell factor (SCF), Flt3L, epidermal growth factor (EGF), keratinocyte growth factor (KGF), fibroblast growth factor (FGF)-1, FGF-2, FGF-9, insulin-like growth factor (IGF)-1, vascular endothelial growth factor (VEGF), β-nerve growth factor (β-NGF), thrombopoietin (TPO), neuropoietin (NPO), platelet-derived growth factor (PDGF)-BB, BM stroma-derived growth factor (SF) 20, interferon (IFN)-γ, IFN-λ2, TNFα, soluble receptor activator of NFκB ligand (sRANKL), a proliferating-inducing ligand (APRIL), TNF ligand superfamily 14 (TNFSF14), transforming growth factor (TGF)-β1, TGF-β2, TGF-β3, activin A, activin B, bone morphogenic protein (BMP)-2, BMP-4, BMP-7, BMP-13, noggin, MIP-2, adiponectin, prolactin, resistin, resistin-like molecule (Relm)-α, and Relm-β. Each cytokine was tested at 10 ng/ml unless otherwise mentioned. 

### Small-scale BM culture system for semi-automated monitoring of neutrophil-DC development

BM cells isolated from C57BL/6 mice (6-8 wks old female) were cultured in round-bottom 96-well plates (4 x 10^5^ cells/200 µl/well) in complete RPMI1640 in the presence or absence of a test cytokine (10 ng/ml). One half of the medium was replaced on Days 2 and 4 with fresh complete RPMI containing the same cytokine at 20 ng/ml. On Day 6, the cells were stained in the 96-well plates with allophycocyanin (APC)-conjugated anti-CD11c monoclonal antibody (mAb), fluorescein isothiocyanate (FITC)-conjugated anti-MHC class II mAb, and phycoerythrin (PE)-conjugated anti-Ly6G mAb and then analyzed for surface expression of CD11c, MHC II, and Ly6G, and for propidium iodide (PI) uptake in a semi-automated fashion with FACSCalibur equipped with a HTP sampler device (BD Biosciences, San Jose, CA) [18]. 

### Conventional BM culture system

BM cells isolated from C57BL/6 mice were cultured in 6-well plates (6 x 10^6^ cells/well) in the presence of selected cytokines. On Days 2 and 4, one half of culture medium was gently replaced with fresh complete RPMI containing the same cytokine. On Day 6, both floating and adherent cells were harvested to examine total cell number, cell viability by PI uptake, and surface expression of CD11c, MHC II, and Ly6G as described previously [16]. 

### Neutrophil culture with BM feeders

Gr-1^high^/CD48^−^ immature neutrophils were sorted from BM cells with >99.5% purity with FACSAria (BD Biosciences). The neutrophils purified from C57BL/6 mice (CD45.2) were then co-cultured in 6-well plates with crude BM feeder cells isolated from B6 SJL mice (CD45.1) in a 1:4 ratio in the presence of selected cytokines. On Day 6, the cells were harvested to analyze surface expression of CD11c, MHC II, and Ly6G within the CD45.2^+^/CD45.1^−^ populations [16]. 

### Thioglycollate-induced peritonitis model

Acute peritonitis was induced in C57BL/6 mice by intraperitoneal (i.p.) injection of 3% thioglycollate (BD Biosciences) (1 ml/animal). To test the impact of IL-4, IL-4 complex was formulated by mixing recombinant IL-4 (PeproTech) with rat mAb against mouse IL-4 (clone BVD4-1D11 from BioXcell, NH) at a 1:5 weight ratio (or 2:1 molar ratio), incubating for 5 minutes on ice, and diluting to an IL-4 concentration of 50 µg/ml in PBS with 1% mouse serum [19–21]. Immediately before thioglycollate injection, 0.2 ml of the above IL-4C formulation (10 µg IL-4/animal) was i.p. injected into mice according to the standard protocol [19–21]. To test the impact of IFNγ, recombinant IFNγ (5 µg/injection/animal) was i.p. injected immediately before and 24 h after thioglycollate administration according to the standard protocol [22,23]. On Day 2, the peritoneal exudate cells (PECs) were collected by washing the peritoneal cavity with 7 ml ice-cold PBS to examine the numbers of neutrophil-DC hybrids after staining for CD11c, MHC II, and Ly6G. The PEC samples were also tested for the ability to capture live bacteria as before [16,17]. Briefly, the PEC samples harvested from the peritonitis lesions were incubated for 60 min with GFP-*E.coli* (MOI = 10), washed extensively, and then analyzed for GFP fluorescence signals within the Ly6G^+^/CD11c^−^ neutrophil, Ly6G^+^/CD11c^+^ hybrid, and Ly6G^-^/CD11c^+^ conventional DC populations by FACSCalibur. All animal experiments were performed in accordance with the NIH guidelines after approval by the Institutional Animal Care and Use Committee of the University of Toledo (Approval Number: 105334). 

### Statistical analyses

All experiments were carried out at least two times to assess reproducibility. All data were processed by GraphPad Prism 5.0 program (GraphPad Software) together with SigmaPlot 10 (Systat Software). Quantitative data are presented as the means ± SD. The statistical significance was assessed based on unpaired Student’s *t*-test with two-tailed distributions or one-way ANOVA with Newma-Keuls multiple comparison test. *P* values smaller than 0.05 were considered to be significant. 

## Results

### Differential impacts of cytokines on development of neutrophil-DC hybrids in BM cultures

We reported recently that neutrophil-DC hybrids expressing the surface phenotype of CD11c^+^/MHC II^+^/Ly6G^+^ emerge in GM-CSF-supplemented BM cultures in a time-dependent fashion with a peak observed on Days 6-9 [16]. Those BM cultures supplemented with GM-CSF also contained neutrophils expressing the phenotype of CD11c^−^/MHC II^-^/Ly6G^+^ and “conventional” DCs expressing the phenotype of CD11c^+^/MHC II^+^/Ly6G^−^. The purpose of the present study was to determine whether other cytokines might regulate the development of neutrophil-DC hybrids. For this purpose, we tested a library of 61 cytokines and chemokines in a systematic fashion. To maximize the time- and cost-efficiency of our screening, we developed a BM micro-culture system, in which BM cells were cultured for 6 days in 96-well plates in the presence of each of the individual cytokines. On Day 6, instead of harvesting the cells for FACS analyses, we directly labeled the cells within the 96-well plates with APC-conjugated anti-CD11c mAb, FITC-conjugated anti-MHC II mAb, and PE-conjugated anti-Ly6G mAb. The samples were subsequently analyzed in the same 96-well plates using the FACS machine equipped with an auto-sampling device. This semi-automated setting enabled us to test a large number of test samples for the frequencies, but not actual numbers, of the above three leukocyte populations emerging in BM micro-cultures. 

**Figure 1:**
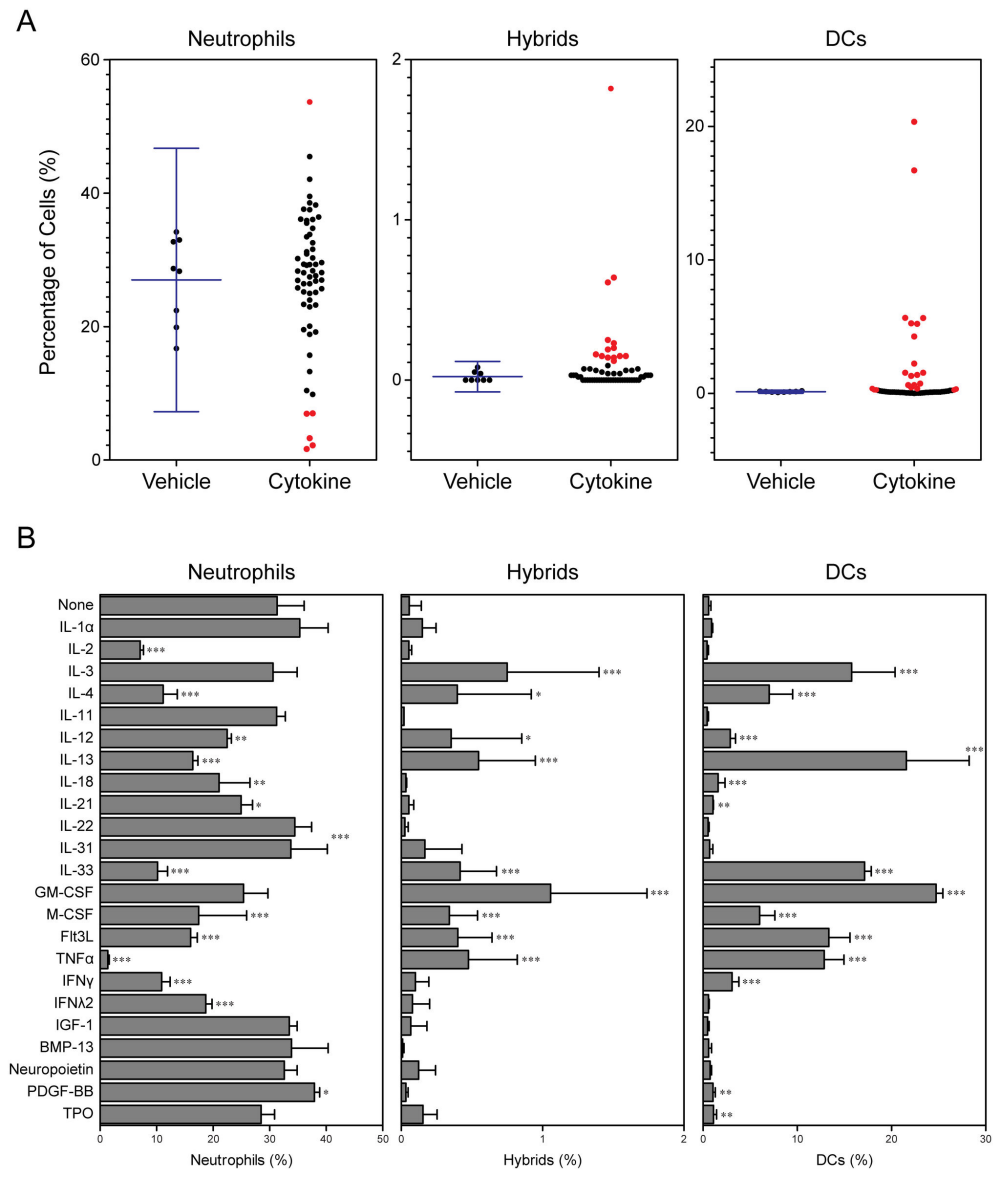
Differential impacts of 61 cytokines on hybrid cell development in crude BM cultures. BM cells freshly procured from adult C57BL/6 mice were cultured in 96-well plates for 6 days in the presence of each of 61 different cytokines (10 ng/ml). The samples were examined in a semi-automated fashion for surface expression of CD11c, MHC II, and Ly6G. (A) Each dot represents the percentage of CD11c^-^/MHC II^-^/Ly6G^+^ neutrophils, CD11c^+^/MHC II^+^/Ly6G^+^ hybrids, or CD11c^+^/MHC II^+^/Ly6G^−^ conventional DCs emerging in BM culture supplemented with a given cytokine. The baseline levels (means ± 3SD) observed in vehicle alone control cultures without added cytokines are shown on the left columns. “Hit” cytokines that increased or decreased the frequencies above or below the baseline levels are indicated in red. The data shown are representative of three independent screening experiments with similar results. (B) The hit cytokines were tested in triplicates at 10 ng/ml. The data shown are the means ± SD of the percentages of neutrophils, hybrids, and conventional DCs. Asterisks indicate statistically significant differences compared with the control cultures without added cytokines (*P < 0.05, **P < 0.01, ***P < 0.001).

 In control cultures supplemented with vehicle alone, only few (less than 0.1%) of the cells expressed all surface markers (i.e., CD11c. MHC II, and Ly6G) of neutrophil-DC hybrids (Figure 1A). Likewise, a relatively minor (~1%) population of the cells expressed the phenotype of conventional DCs (i.e., CD11c^+^/MHC II^+^/Ly6G^−^) in those control cultures without added cytokines, although CD11c^−^/MHC II^-^/Ly6G^+^ neutrophils were found at high frequencies (20-30%). Based on the frequencies of each leukocyte population found in the control cultures, we calculated the means ± 3SD values as the baselines levels (error bars in Figure 1A). A total of 23 cytokines (indicated in red in Figure 1A) were found to increase or reduce the percentages of hybrids, conventional DCs, and/or neutrophils above or below the baseline levels. These cytokines that produced similar outcomes in three independent screening experiments were registered as “hits.” 

 In the second screening, the hit cytokines were examined in triplicates in the above BM micro-culture system (Figure 1B). Once again, control cultures without added cytokines contained <0.1% neutrophil-DC hybrids, ~1% conventional DCs, and 20-30% neutrophils. As expected, GM-CSF significantly (P<0.001) promoted the generation of both hybrids and conventional DCs. Both populations also emerged in elevated levels in BM cultures supplemented with other cytokines, such as IL-3, IL-4, IL-13, IL-33, M-CSF, and Flt3L. Interestingly, many of these cytokines, except GM-CSF and IL-3, reduced the frequencies of neutrophils in BM cultures. GM-CSF appeared to be the most potent cytokine promoting the generation of neutrophil-DC hybrids at the tested concentration (10 ng/ml). 

**Figure 2:**
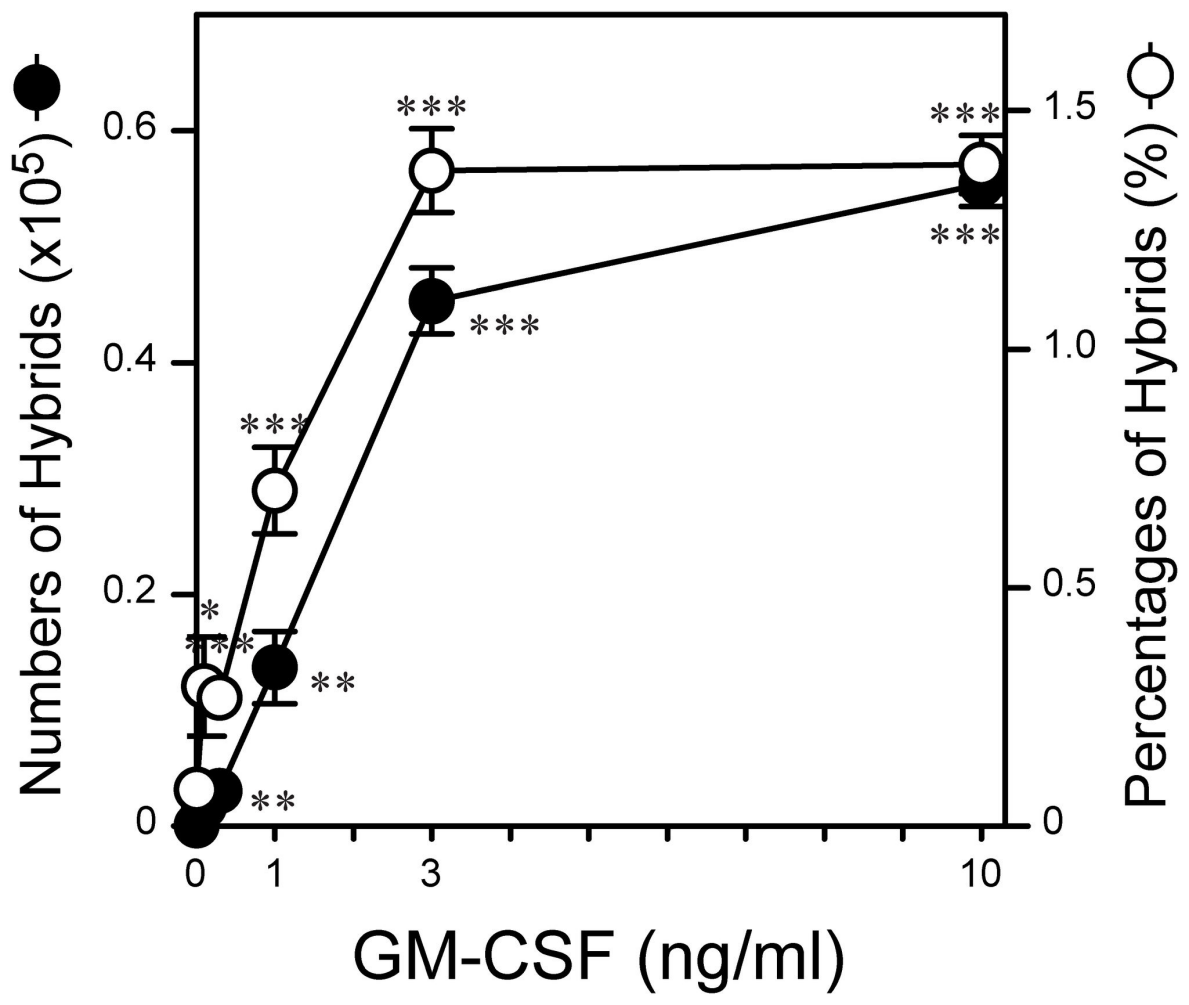
Hybrid cell development induced by GM-CSF alone. BM cells were cultured in 6-well plates for 6 days in the presence of GM-CSF at the indicated concentrations. The samples were harvested and then examined for surface expression of CD11c, MHC II, and Ly6G. The data shown are the absolute numbers (closed circles) and the percentages (open circles) of CD11c^+^/MHC II^+^/Ly6G^+^ neutrophil-DC hybrids (means ± SD from triplicate samples). Asterisks indicate statistically significant differences compared with the control cultures without added GM-CSF (*P < 0.05, **P < 0.01, ***P < 0.001). Data are representative of at least three independent experiments with similar results.

 To test the impact of GM-CSF more closely, we cultured BM cells in relatively large quantities in 6-well plates in the presence of GM-CSF added at graded concentrations. On Day 6, we harvested the cells and counted the total cell numbers recovered from the cultures. The harvested cells were then stained for CD11c, MHC II, and Ly6G in test tubes and analyzed by FACS manually. This traditional approach enabled us to determine not only the frequencies of CD11c^+^/MHC II^+^/Ly6G^+^ hybrids, CD11c^+^/MHC II^+^/Ly6G^−^ conventional DCs, and CD11c^−^/MHC II^-^/Ly6G^+^ neutrophils, but also the actual numbers of individual leukocyte populations emerging in the BM cultures. GM-CSF was found to increase both the percentage and actual number of neutrophil-DC hybrids in a dose-dependent manner (Figure 2). Relatively low concentrations (0.3-1 ng/ml) were sufficient to augment hybrid cell generation significantly (P<0.01), whereas higher concentrations (3-10 ng/ml) were required for maximal effects. 

### Opposing impacts of IL-4 versus IFNγ on the in vitro generation of neutrophil-DC hybrids

**Figure 3:**
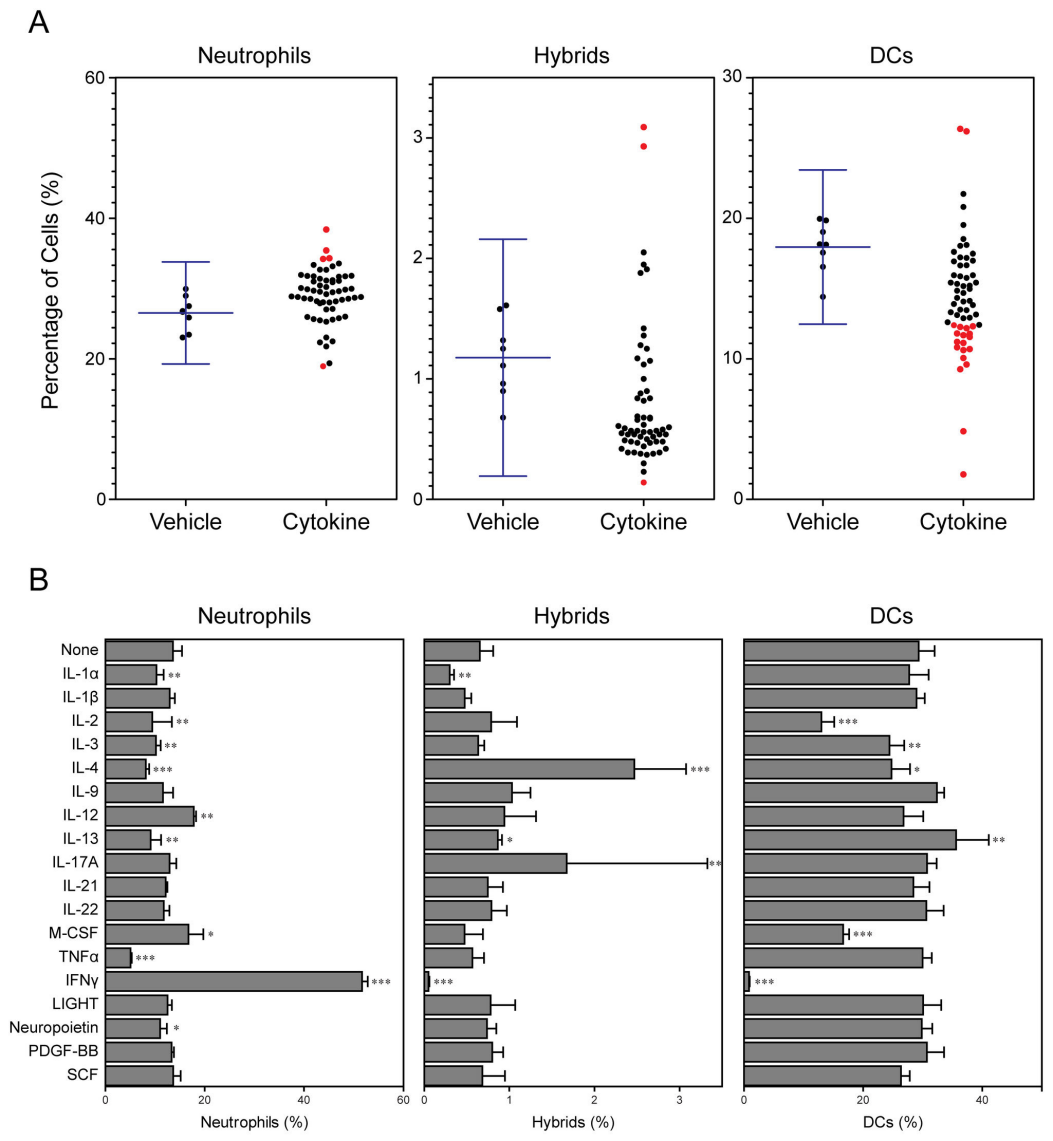
Differential impacts of cytokines tested in combination with GM-CSF. BM cells were cultured in 96-well plates for 6 days with each of 60 different cytokines (10 ng/ml) in the presence of 10 ng/ml GM-CSF. The samples were examined in a semi-automated fashion for surface expression of CD11c, MHC II, and Ly6G. (A) Each dot represents the percentage of CD11c^-^/MHC II^-^/Ly6G^+^ neutrophils, CD11c^+^/MHC II^+^/Ly6G^+^ hybrids, or CD11c^+^/MHC II^+^/Ly6G^−^ conventional DCs emerging in BM culture supplemented with a given cytokine. The baseline levels (means ± 3SD) observed in the control cultures with GM-CSF alone are shown on the left columns. “Hit” cytokines that increased or decreased the frequencies above or below the baseline levels are indicated in red. The data shown are representative of three independent screening experiments with similar results. (B) The hit cytokines were tested in triplicates at 10 ng/ml, again, in the presence of 10 ng/ml GM-CSF. The data shown are the means ± SD of the percentages of neutrophils, hybrids, and conventional DCs. Asterisks indicate statistically significant differences compared with the control cultures with GM-CSF alone (*P < 0.05, **P < 0.01, ***P < 0.001).

 Having identified GM-CSF as a potent cytokine promoting hybrid cell generation, we next screened the same cytokine library in the presence of added GM-CSF in the BM micro-culture system (Figure 3A). A total of 18 cytokines were found to increase or reduce the percentages of hybrids, conventional DCs, and/or neutrophils above or below the baseline levels (means ± 3SD) observed with GM-CSF alone (Figure 3A). GM-CSF-dependent hybrid cell formation was enhanced by two cytokines (IL-4 and IL-17A) and suppressed by a single cytokine (IFNγ). Again, these hit cytokines were examined in triplicates in the second screening (Figure 3B). IL-4 induced significant (P < 0.001) and marked (>3-fold) augmentation of GM-CSF-induced hybrid cell generation. By contrast, IFNγ induced significant (P < 0.001) and marked (>80%) inhibition of GM-CSF-induced hybrid cell development. 

**Figure 4:**
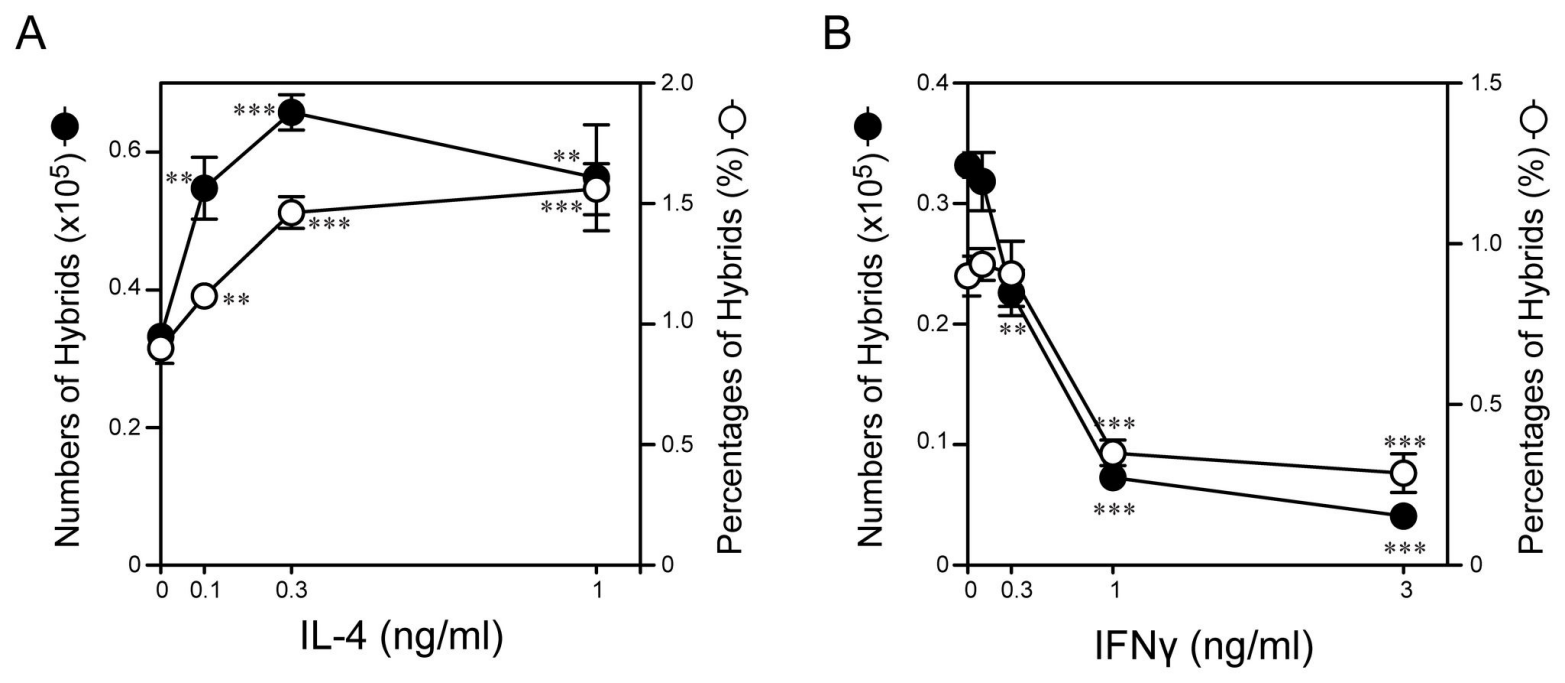
Opposing impacts of IL-4 and IFNγ on GM-CSF-induced hybrid cell development. BM cells were cultured in 6-well plates for 6 days in the presence of 10 ng/ml GM-CSF plus IL-4 (A) or IFNγ (B) added at the indicated concentrations. The samples were harvested and then examined for surface expression of CD11c, MHC II, and Ly6G. The data shown are the absolute numbers (closed circles) and the percentages (open circles) of CD11c^+^/MHC II^+^/Ly6G^+^ neutrophil-DC hybrids (means ± SD from triplicate samples). Asterisks indicate statistically significant differences compared with the control cultures without added GM-CSF (*P < 0.05, **P < 0.01, ***P < 0.001). Data are representative of at least four independent experiments.

 To confirm these observations, we added IL-4 or IFNγ at graded concentrations to traditional BM cultures in 6-well plates supplemented with 10 ng/ml GM-CSF. Addition of IL-4 at 1 ng/ml induced significant (P < 0.001) and substantial (~2-fold) increases in both the percentage and actual number of CD11c^+^/MHC II^+^/Ly6G^+^ hybrids above the levels observed in the control culture with GM-CSF alone (Figure 4A). By marked contrast, IFNγ at 1 ng/ml induced significant (P < 0.001) and marked (60-70%) inhibition of GM-CSF-induced hybrid cell generation (Figure 4B). 

**Figure 5:**
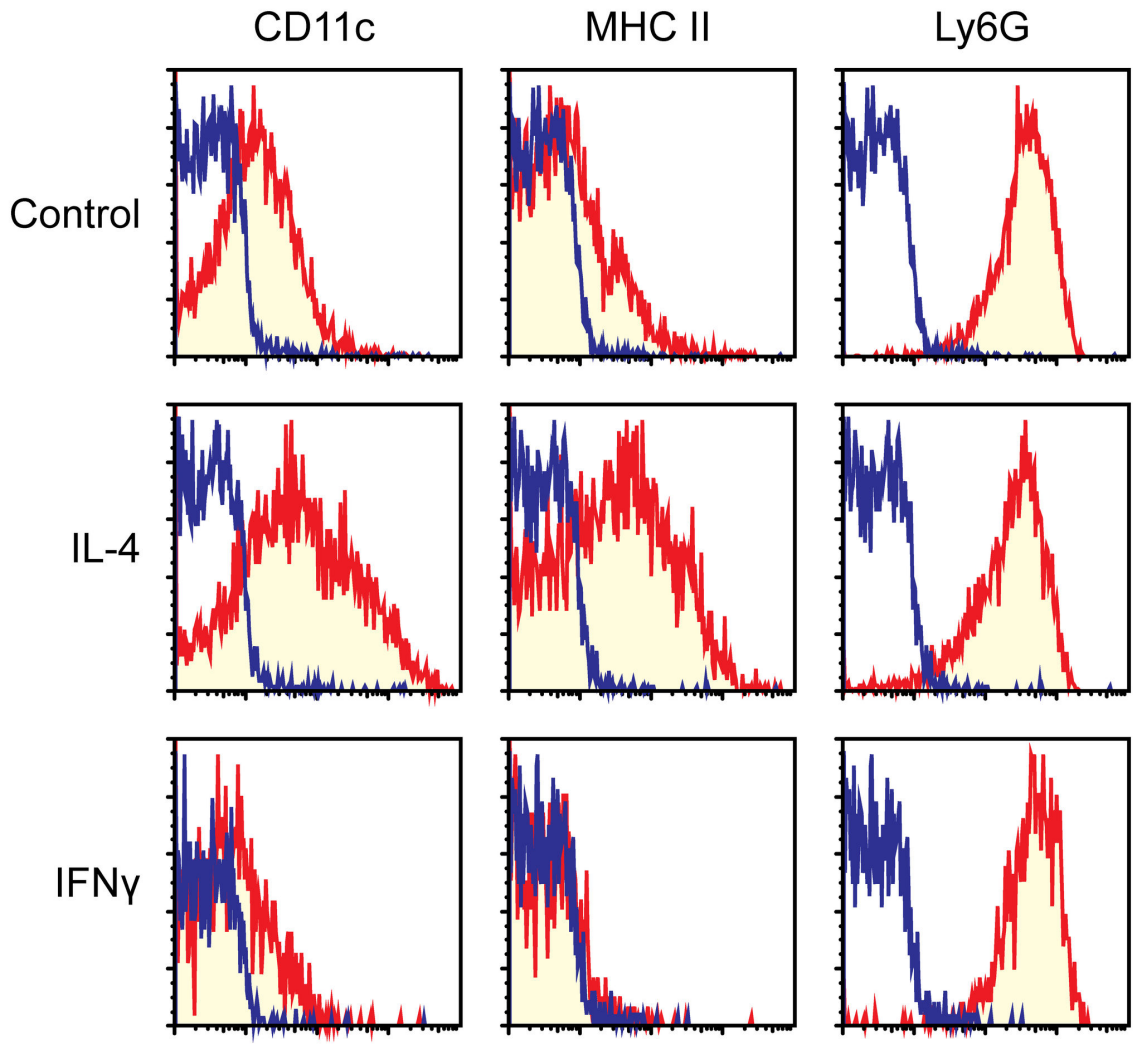
Direct impacts of IL-4 and IFNγ on GM-CSF-induced transdifferentiation of neutrophils. Gr-1^high^/CD48^−^ immature neutrophils purified from BM of C57BL/6 mice (CD45.2) were co-cultured for 6 days with BM feeder cells from B6 SJL mice (CD45.1) in the presence of 10 ng/ml GM-CSF alone (top panels), GM-CSF and 10 ng/ml IL-4 (middle panels), or GM-CSF and 10 ng/ml IFNγ (bottom panels). The samples were examined for surface expression of the indicated markers within the CD45.2^+^/CD45.1^−^ gated populations. Blue lines indicate staining profiles with isotype-matched control IgG. Data are representative of at least four independent experiments.

 All the above experiments were performed with crude BM cells as starting populations. In the next experiments, we sought to directly test the impacts of IL-4 versus IFNγ on the ability of neutrophils to undergo transdifferentiation into neutrophil-DC hybrids. For this purpose, we isolated Gr-1^high^/CD48^−^ immature neutrophils from BM of C57BL/6 mice (CD45.2) with >99.5% purity – this population uniformly expressed the surface phenotype of Ly6G^+^/Ly6C^+^/7/4^+^/CD11b^+^/CD11c^−^/MHC II^−^ and displayed a typical morphology of mouse band cells characterized by ring-shaped nuclei [16]. Those purified neutrophils were then co-cultured with crude BM feeder cells isolated from B6 SJL mice (CD45.1) in the presence of GM-CSF alone, GM-CSF plus IL-4, or GM-CSF plus IFNγ. We employed this co-culture system because long-term survival of highly purified neutrophils can be enhanced by the presence of BM feeder cells [16]. On Day 6, we harvested the cells to examine surface expression of CD11c, MHC II, and Ly6G within the CD45.2^+^/CD45.1^−^ populations (Figure 5). In the culture supplemented with GM-CSF alone, neutrophils acquired surface expression of CD11c (a DC marker), while maintaining uniform expression of Ly6G (a neutrophil marker). MHC II expression also became detectable in some of these cells. Importantly, addition of IL-4 markedly enhanced surface expression of both CD11c and MHC II, whereas added IFNγ produced opposing outcomes. Neither IL-4 nor IFNγ affected surface expression of Ly6G. Based on these in vitro observations, we concluded that GM-CSF-induced neutrophil transdifferentiation into hybrid cells can be counter-regulated by IL-4 and IFNγ. 

### In vivo emergence of neutrophil-DC hybrids at inflammatory sites can be differentially regulated by altering local cytokine milieus

**Figure 6:**
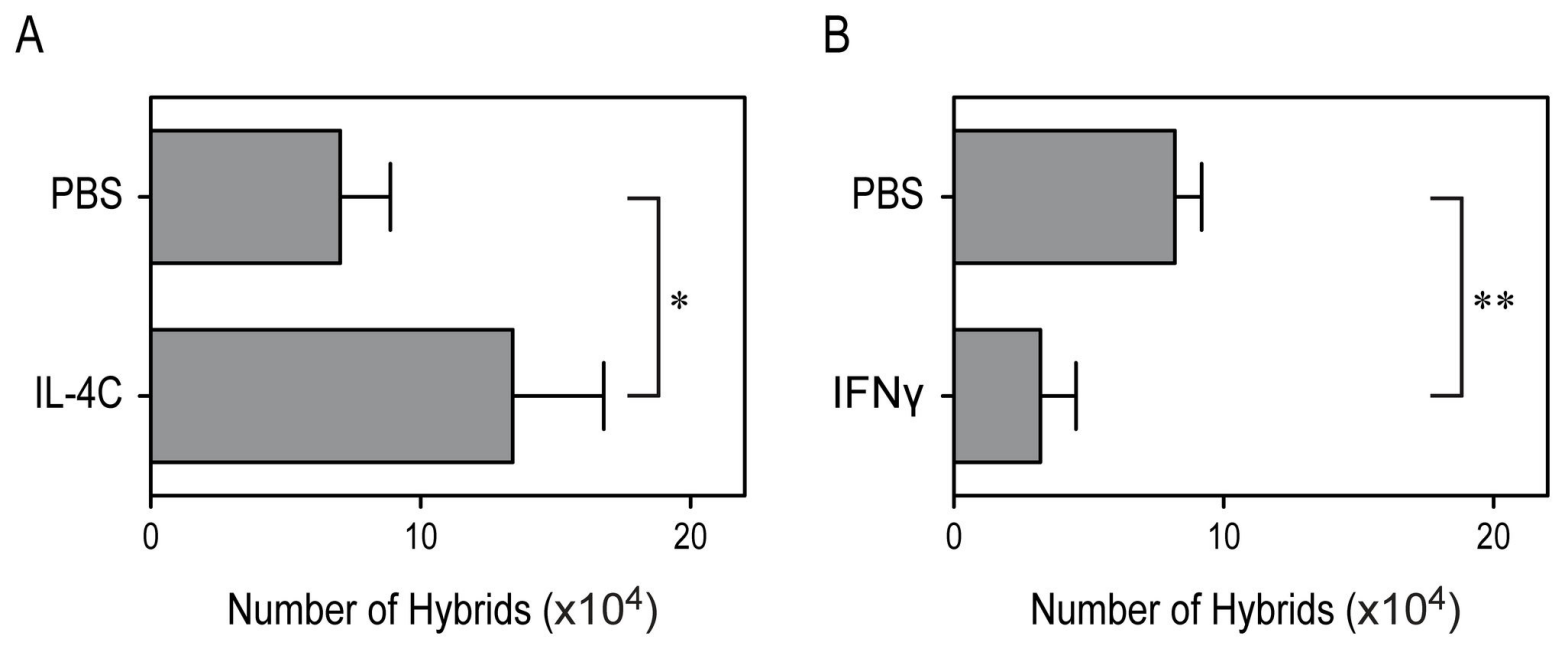
Cytokine-dependent regulation of in vivo emergence of hybrid cells. C57BL/6 mice (3 animals/panel/experiment) received i.p. injection of thioglycollate. (A) Immediately before thioglycollate injection, freshly prepared IL-4C formulation (10 µg IL-4/animal) or PBS alone was i.p. injected into those mice. (B) Recombinant IFNγ (5 µg/injection/animal) or PBS alone was i.p. injected immediately before and 24 h after thioglycollate administration. PEC samples harvested on Day 2 were analyzed for the numbers of CD11c^+^/MHC II^+^/Ly6G^+^ neutrophil-DC hybrids. Asterisks indicate statistically significant differences compared with the control panel receiving PBS alone (*P < 0.05, **P < 0.01). Data are representative of four independent in vivo experiments.

 A key question concerned whether in vivo generation of neutrophil-DC hybrids can be counter-regulated by these cytokines. To test this, we employed thioglycollate-induced peritonitis as a model of inflammation because CD11c^+^/MHC II^+^/Ly6G^+^ hybrids become readily detectable in the PEC samples [17]. To extend the biological half-life of IL-4, we administered the IL-4C formulation composed of recombinant IL-4 and anti-IL-4 mAb according to the standard protocol [19–21]. A single i.p. injection of IL-4C resulted in a significant (P < 0.05) and substantial (roughly 2-fold) increase in the number of neutrophil-DC hybrids recovered in the PEC samples (Figure 6A). In the next set of experiments, we tested the in vivo impact of IFNγ by administering recombinant IFNγ according to the standard protocol [22,23]. Two repeated i.p. injections of IFNγ produced a significant (P < 0.01) and noticeable (>60%) reduction in hybrid cell recovery (Figure 6B). These in vivo findings support our conclusion that generation of neutrophil-DC hybrids at inflammatory sites can be counter-regulated by altering local cytokine milieus.

**Figure 7:**
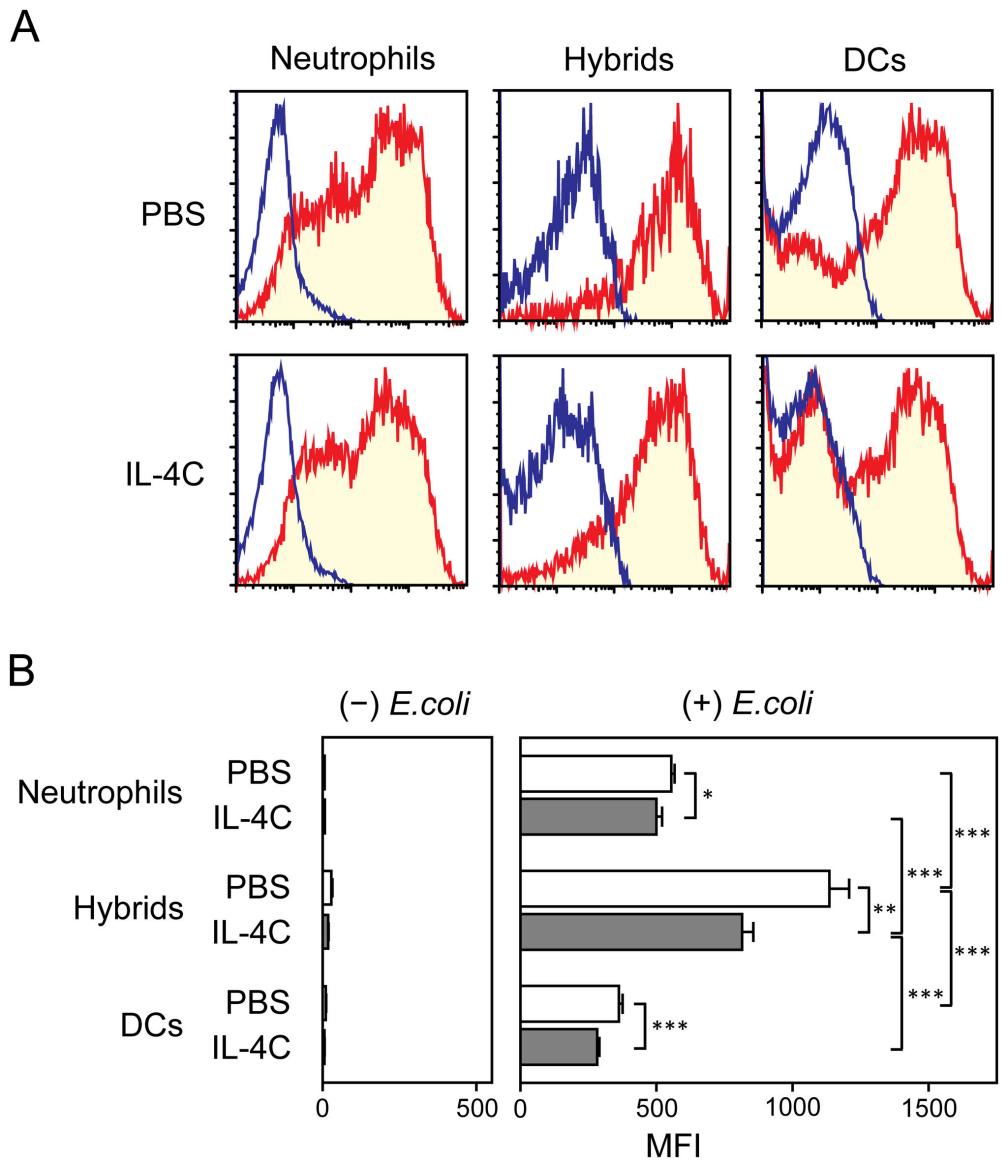
Endocytotic activity of neutrophil-DC hybrids emerging in IL-4C-treated animals. Immediately after i.p. injection of IL-4C or PBS alone, C57BL/6 mice (3 animals/panel) received i.p. injection of thioglycollate. PEC samples harvested on Day 2 were incubated for 60 min at 37°C with live *E.coli* expressing GFP and then examined for GFP fluorescence signals within the Ly6G^+^/CD11c^−^ neutrophil, Ly6G^+^/CD11c^+^ hybrid, and Ly6G^-^/CD11c^+^ conventional DC populations. (A) Data shown are representative histograms after incubation in the presence (red) or absence (blue) of GFP-*E*.*coli*. (B) Data shown are the means ± SD of MFI values from three animals. Asterisks indicate statistically significant differences (*P < 0.05, **P < 0.01; ***P < 0.001).

 We have observed previously that neutrophil-DC hybrids generated in vitro in GM-CSF-supplemented BM cultures as well as the hybrids purified from peritonitis lesions exhibit a potent ability to capture bacteria [16,17]. Thus, we next sought to determine whether those neutrophil-DC hybrids emerging in the presence of IL-4C would exhibit this functionality. We administered IL-4C (or PBS alone) immediately before thioglycollate injection, harvested PEC samples on Day 2, and tested their ability to capture GFP-*E.coli*. Consistent to our previous observations, neutrophil-DC hybrids harvested from control mice receiving PBS alone showed effective bacterial uptake as assessed by measuring GFP fluorescence signals (Figure 7A, upper middle panel). Two other leukocyte populations (i.e., neutrophils and conventional DCs) in the same PEC samples also showed significant bacterial uptake. Importantly, strong GFP signals were detected in the hybrids harvested from IL-4C-treated mice (Figure 7A, lower middle panel). Quantitative analyses based on the mean fluorescence intensity (MFI) values unveiled significant, albeit modest, difference in bacterial uptake by neutrophil-DC hybrids between the IL-4C-treated panel and PBS-treated control panel (Figure 7B). Nevertheless, hybrid cells showed the most efficient bacterial uptake among the three tested leukocyte populations in both PBS- and IL-4C-treated panels. 

**Figure 8:**
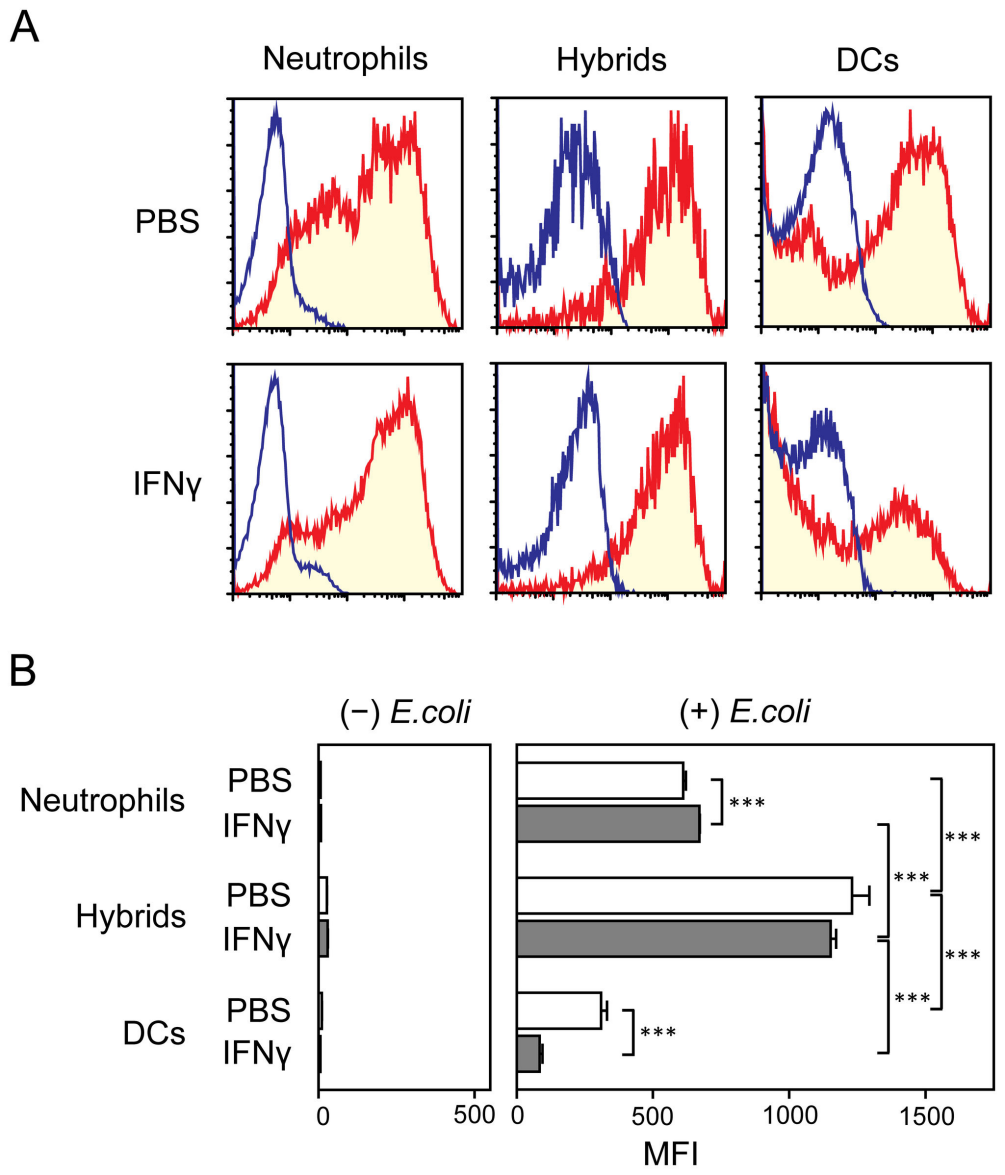
Endocytotic activity of neutrophil-DC hybrids emerging in IFNγ-treated animals. C57BL/6 mice (3 animals/panel) received i.p. injections of recombinant IFNγ or PBS alone immediately before and 24 h after thioglycollate treatment. PEC samples harvested on Day 2 were incubated for 60 min at 37°C with live *E.coli* expressing GFP and then examined for GFP fluorescence signals within the Ly6G^+^/CD11c^−^ neutrophil, Ly6G^+^/CD11c^+^ hybrid, and Ly6G^-^/CD11c^+^ conventional DC populations. (A) Data shown are representative histograms after incubation in the presence (red) or absence (blue) of GFP-*E*.*coli*. (B) Data shown are the means ± SD MFI values from three animals. Asterisks indicate statistically significant differences (*P < 0.05, **P < 0.01; ***P < 0.001).

 We next examined bacterial uptake by neutrophil-DC hybrids harvested from IFNγ-treated mice – they were found to be comparable to those from PBS-treated mice in their capacity to capture GFP-*E.coli* (Figure 8). Corroborating with the previous reports [24,25], IFNγ treatment augmented bacterial uptake by neutrophils, while suppressing the same functionality of conventional DCs. In summary, our findings demonstrate that IL-4C and IFNγ can counter-regulate the generation of neutrophil-DC hybrids without markedly impairing their functionality. 

## Discussion

Through unbiased screening of 61 cytokines, we have identified IL-4 and IFNγ as potent regulators of the development of neutrophil-DC hybrids. To recapitulate the essence of our findings, IL-4 was found to augment GM-CSF-dependent generation of CD11c^+^/MHC II^+^/Ly6G^+^ hybrids in BM cultures. Added IL-4 also promoted transdifferentiation of purified immature neutrophils into hybrids in the presence of GM-CSF and BM feeder cells. Most importantly, we were able to expand the number of neutrophil-DC hybrids emerging at inflammatory sites by administering IL-4C. By marked contrast, IFNγ produced completely opposing outcomes in each of these assays. Thus, we propose that IL-4 and IFNγ can counter-regulate the generation of neutrophil-DC hybrids, a recently discovered leukocyte population showing dual functionality of professional phagocytes and APCs. 

It is known that human neutrophils begin to express several DC markers when cultured in the presence of selected cytokines in various combinations. For example, the combination of GM-CSF, IL-4, and TNFα induced the expression of MHC II, CD40, CD86, CD1a, CD1b, and CD1c by neutrophil-committed precursors isolated from leukemia patients receiving G-CSF treatments [7]. The combination of GM-CSF and IFNγ has been employed by several groups to trigger MHC II, CD80, CD83, and CD86 expression by neutrophils isolated from healthy individuals [6,15,26–28]. Interestingly, human neutrophils cultured with TNFα and IFNγ displayed CD83 on the surface and expressed mRNA for a DC-specific CC-chemokine receptor CCR6 [8]. Exposure to IL-15 induced MHC II and CD86 expression by human neutrophils [9]. Thus, human neutrophils possess the potential to express various DC markers in culture depending upon the cytokine milieus. With regard to in vivo relevance, unusual neutrophils expressing DC markers have been detected in synovial fluid samples from patients with rheumatoid arthritis [12] and in peripheral blood samples from patients with Wegener’s granulomatosis [14,15,29]. In mice, neutrophils isolated from chronic colitis lesions have been shown to express MHC II and CD86 and exhibit an APC capacity to present peptide antigen to CD4 T cells [11]. We reported recently that a “hybrid” leukocyte population displaying phenotypic and functional properties of both neutrophils and DCs becomes readily detectable in experimentally induced inflammatory lesions in mice [17]. Moreover, FACS-purified murine neutrophils began to show the dual properties of neutrophil-DC hybrids when cultured with GM-CSF [16]. In essence, the hybrid cells can function as professional phagocytes by efficiently capturing soluble and particulate materials and live bacteria from extracellular spaces. At the same time, they can also function as APCs by presenting exogenous antigens to CD4 T cells [16,17]. However, mechanisms regulating neutrophil transdifferentiation into hybrids remain mostly unknown. 

The objective of the present study was to identify the cytokines capable of regulating the formation of hybrids. We tested a total of 61 cytokines individually and in combination with GM-CSF for their impacts on the development of neutrophil-DC hybrids as defined by dual expression of DC markers (CD11c and MHC II) and a neutrophil marker (Ly6G). When added individually to crude BM cultures, GM-CSF markedly promoted the emergence of CD11c^+^/MHC II^+^/Ly6G^+^ hybrids (Figure 1). IL-3 also supported the generation of hybrids – it should be stated that Ly6G is expressed by neutrophil-derived hybrids, but not by monocyte-derived DCs [16]. Thus, IL-3 did not simply cause DC-directed differentiation of monocytes present in BM cell preparations. Interestingly, GM-CSF and IL-3 have both been reported to sustain the survival of mouse neutrophils by counter-acting with the pro-apoptotic signaling cascades triggered by Bcl-2 family member Bim [30]. Thus, it is tempting to speculate that GM-CSF and IL-3 promote formation of neutrophil-DC hybrids by extending the life-span of neutrophils and by triggering their DC-directed transdifferentiation. Although relatively low concentrations (0.3-1 ng/ml) of GM-CSF were sufficient to induce the generation of hybrid cells at detectable levels, higher concentrations (3-10 ng/ml) were required to induce the maximal generation (Figure 2). When combined with 10 ng/ml GM-CSF, IL-4 was found to markedly augment hybrid cell generation (Figure 3). In dose-dependency experiments, IL-4 at 1-3 ng/ml induced the maximal (2-fold) augmentation of GM-CSF-induced hybrid cell formation (Figure 4A). By marked contrast, IFNγ was found to inhibit GM-CSF-induced hybrid cell development almost completely at 1-3 ng/ml (Figure 4B). These observations did not appear to be in complete agreement with the previous reports showing that human neutrophils acquired surface expression of MHC II and co-stimulatory molecules when cultured with GM-CSF, IL-4, and TNFα[7], as well as with GM-CSF and IFNγ [6,15,26,27]. Thus, we directly compared the impacts of IL-4 versus IFNγ on GM-CSF-induced transdifferentiation of FACS-purified neutrophils (Figure 5). The starting neutrophil population uniformly expressed Ly6G at high levels, whereas neither CD11c nor MHC II was detectable on the surfaces. When cultured with GM-CSF alone (in the presence of BM feeder cells), some neutrophils acquired surface expression of CD11c and MHC II. Addition of IL-4 to these GM-CSF-supplemented neutrophil cultures markedly elevated the expression levels of both CD11c and MHC II. Once again, added IFNγ almost completely abolished GM-CSF-induced acquisition of CD11c and MHC II by neutrophils. Moreover, we confirmed these observations in vivo in the thioglycollate-induced peritonitis model (Figure 6). Administration of IL-4C resulted in a roughly 2-fold increase in the number of neutrophil-DC hybrids recovered from the inflammatory lesions. By contrast, the emergence of hybrid cells was significantly inhibited by IFNγ administration. Importantly, those hybrids harvested from the mice treated with IL-4C or with IFNγ exhibited a potent ability to incorporate live bacteria (Figures 7 and 8). Taken together, these observations have unveiled previously unrecognized abilities of IL-4 and IFNγ to regulate the development of neutrophil-DC hybrids in opposing directions. 

Our findings are striking because IFNγ and IL-4 are known to drive macrophage activation into two opposing directions known as classical (or M1) and alternative (or M2) states of activation, respectively [31–34]. In essence, M1 macrophages, which produce nitric oxide, reactive oxygen species, and proinflammatory cytokines, are considered to promote Th1-biased immunity against intracellular pathogens. By contrast, M2 macrophages, which produce extracellular matrix components, are thought to play important roles in tissue repair. Interestingly, the two cytokines also counter-regulate monocyte differentiation. Monocytes stimulated with GM-CSF plus IL-4 differentiate into DCs, whereas addition of IFNγ to these cultures skews their differentiation into macrophages [35]. Here we demonstrate for the first time that neutrophil transdifferentiation into hybrid cells is counter-regulated by the same two cytokines. Thus, IFNγ and IL-4 appear to represent the key cytokines that determine the fate and functionality of all three phagocyte populations, i.e., neutrophils, monocytes, and macrophages. 

The BM micro-culture system in 96-well plates combined with in situ immunofluorescence staining and semi-automated FACS analysis enabled us to test a relatively large number of cytokines in a time- and cost-efficient manner. On the other hand, the extent of sample-to-sample variations was relatively large with this assay system especially for neutrophil-DC hybrids due to their low frequencies (Figures 1A and 3A). We were able to markedly diminish the level of variations in the second screening by culturing BM cells in 6-well plates, harvesting the cells for staining, and manually analyzing a relatively large number of cells (Figures 2 and 4). Our two-step approaches (i.e., the first screening with BM micro-culture system followed by the second screening with more traditional BM culture system) may be used for large-scale screening of small compounds to develop a unique class of drugs designed to regulate the generation of neutrophil-DC hybrids. False negative results in the first screening (due to variations) would be a potential pitfall of this screening strategy. 

It is important to point out the limitations of the present study. First, in addition to GM-CSF, several cytokines (IL-3, IL-13, IL-33, M-CSF, Flt3L, and TNFα) were found to promote the generation of neutrophil-DC hybrids above the baseline level. It remains to be determined whether IL-4 and/or IFNγ affect hybrid cell development induced by these cytokines. Second, we only tested bacterial uptake as a functional parameter. More comprehensive analyses are required to define the functionality of neutrophil-DC hybrids emerging in the presence of IL-4 and/or IFNγ. Finally, we tested the impact of exogenously added cytokines, but not the contribution of endogenously produced cytokines. Studies are in progress in our laboratory to determine which endogenous cytokine(s) are required for the generation of neutrophil-DC hybrids at inflammatory sites. Nevertheless, we believe that the present study now provides a framework for our understanding of mechanisms that regulate transdifferentiation of neutrophils into hybrids. 
